# Primary Vesical Amyloidosis Masquerading as Vesical Calculus in a Retroviral Positive Patient

**DOI:** 10.7759/cureus.3828

**Published:** 2019-01-04

**Authors:** Vijay Ganapathy, Chellappa Vijayakumar, Vilvapathy Senguttuvan Karthikeyan, J Srinivas, Sadhanandham Shrinuvasan

**Affiliations:** 1 Urology, Indira Gandhi Medical College & Research Institute, Puducherry, IND; 2 Surgery, Jawaharlal Institute of Postgraduate Medical Education and Research, Puducherry, IND; 3 Urology, Institute of Nephro Urology, Bengaluru, IND; 4 Radiology, Sri Lakshmi Narayana Institute of Medical Science, Puducherry, IND

**Keywords:** genitourinary, vesical calculus, renal amyloidosis, retro positive, bladder cancer

## Abstract

The common causes of irritative voiding symptoms in women include cystitis, vesical calculi, carcinoma bladder and neurologic disorders. Isolated primary vesical amyloidosis (VA) is a rare cause of irritative voiding symptoms.

A 50-year-old female, a known case of retroviral disease but not on anti-retroviral therapy, presented with right flank and suprapubic pain for six months, worsening over the past 15 days with dysuria. She also presented with increased frequency of micturition with nocturia and urgency for the same duration. She had no hematuria, other lower urinary tract symptoms or fever. Clinical examination revealed suprapubic tenderness. Ultrasonogram (USG) revealed 1.7 cm vesical calculus. Cystoscopy revealed three spiky vesical calculi. There was a fluffy lesion with mucosal edema over the right lateral wall in the region of the right ureteric orifice, which was biopsied. Biopsy showed fragments of urothelial mucosa with focal areas of ulceration. The underlying stroma was edematous with amorphous pale eosinophilic acellular deposits. Congo red stain showed apple-green birefringence under polarized microscopy suggestive of amyloid. Sections were negative for dysplasia, granulomas or malignancies.

VA presents with intermittent gross hematuria in up to 77% patients and irritative voiding symptoms in 23% patients. VA is an uncommon differential diagnosis of cancer urinary bladder, with less than 200 cases reported in the literature. Hence we report this case to highlight that primary VA should also be considered in the evaluation of irritative voiding symptoms.

## Introduction

Amyloidosis is defined by deposition of extracellular, hyaline, proteinaceous and amorphous material in various parts of the body. It can be classified into immunocyte dyscrasia (primary type) and complication of an underlying chronic disease (secondary type) [1]. Amyloidosis in genitourinary system is rare. However, amongst the various sites in the genitourinary system, urinary bladder is the most commonly affected organ [2]. Both primary and secondary amyloidosis may involve the bladder, isolated primary vesical amyloidosis (VA) is more common than secondary one [2]. These lesions commonly confused with malignancy. Painless gross haematuria is the main presenting symptoms in most of the patients [2-4]. Accurate diagnosis depends on the biopsy of the lesion and immunohistochemical (IHC) studies identifying the amyloid type [2,3]. Both sexes are equally affected between the sixth and seventh decade of life.

## Case presentation

**Figure 1:**
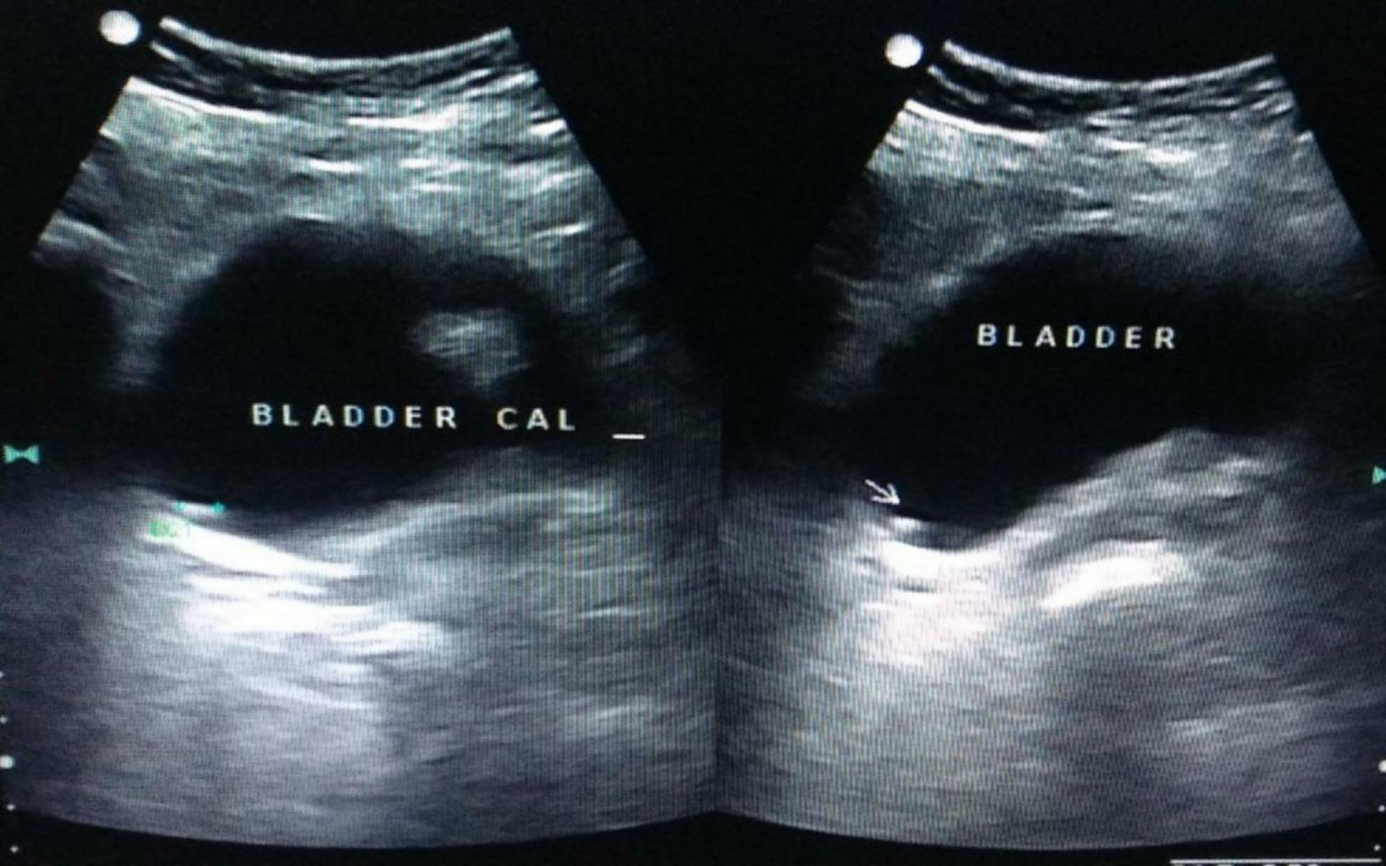
Ultrasonogram KUB (kidney urinary bladder) - Revealed 1.2 cm right upper pole renal calculus with 1.7 cm vesical calculus (arrow).

A 50-year-old female, a known case of retroviral disease but not on anti-retroviral therapy, presented with right flank pain and suprapubic pain for six months which worsened over the past 15 days with dysuria and increased frequency of micturition with nocturia and urgency. She had no hematuria, other lower urinary tract symptoms or fever. Clinical examination revealed suprapubic and right iliac fossa tenderness. She was anemic (Haemoglobin 7.6 g/dL) which was corrected with packed red blood cell transfusions. Her renal function test was normal. Ultrasonogram (USG) of kidney ureter bladder (KUB) revealed 1.2 cm right upper pole renal calculus with 1.7 cm vesical calculus (Figure 1).

**Figure 2:**
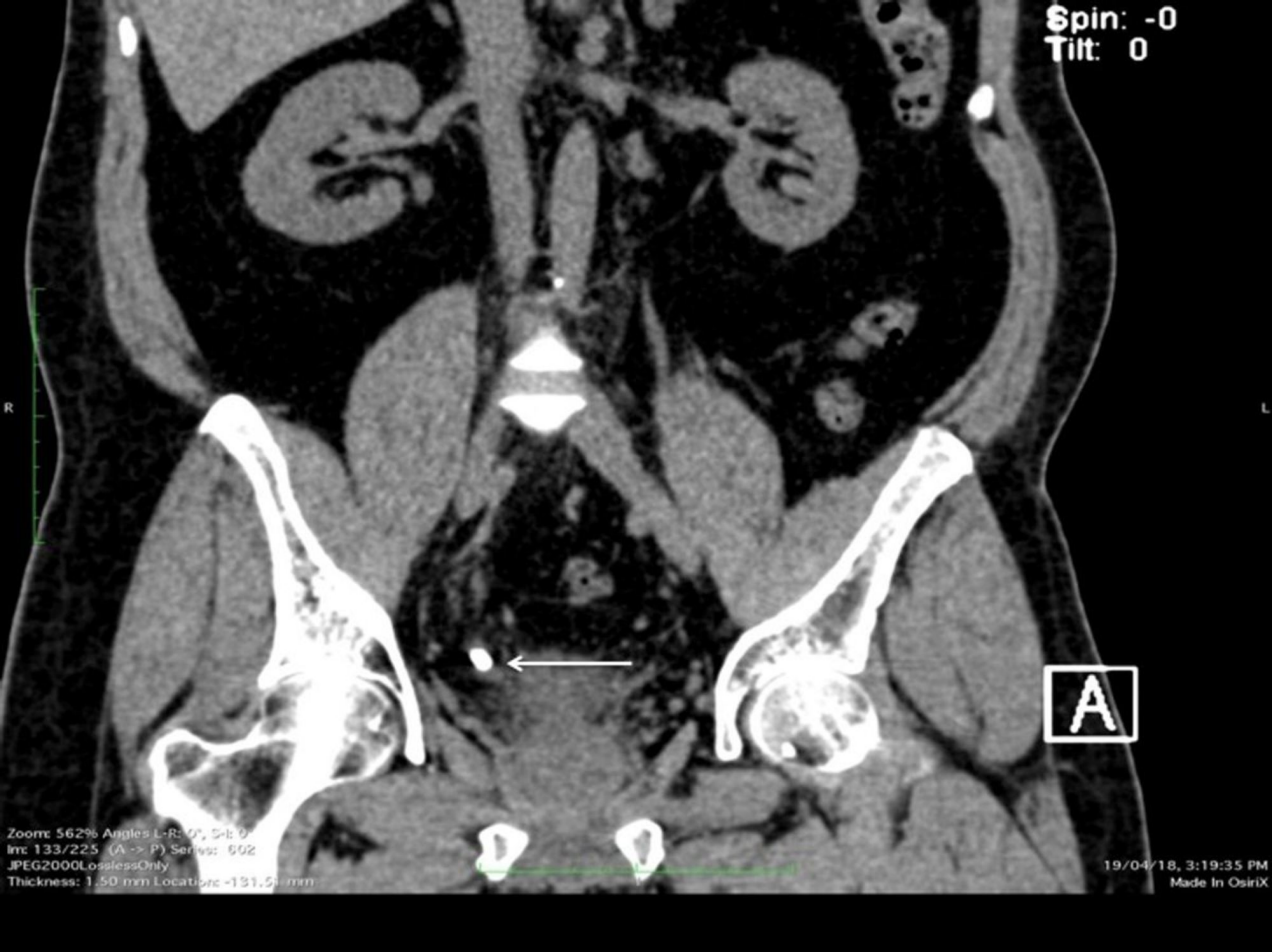
CT KUB (coronal view) - Revealed bladder calculi (arrow). CT: Computed tomography; KUB: Kidney ureter bladder.

**Figure 3:**
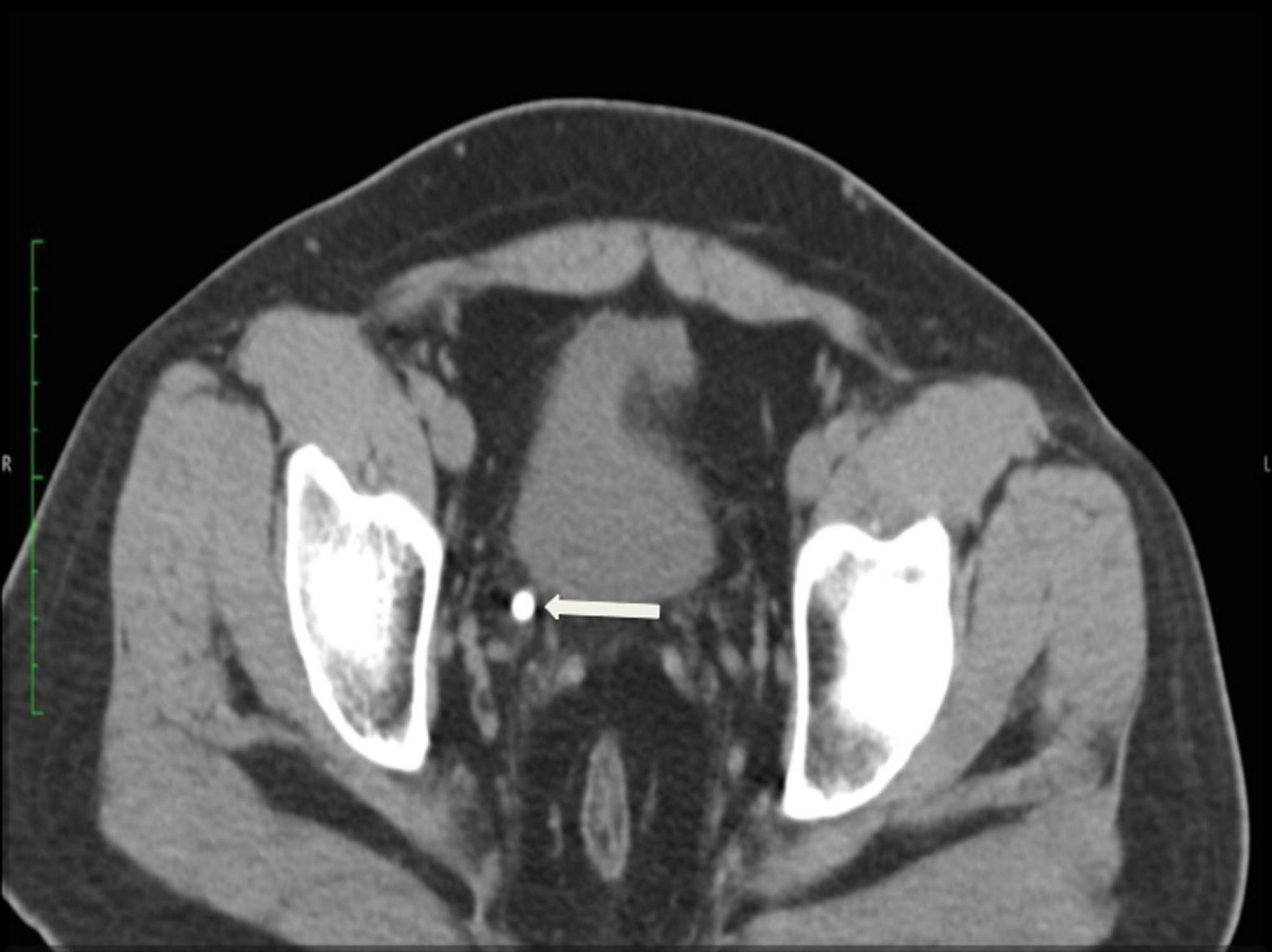
CT KUB (axial view) - Revealed bladder calculi (arrow). CT: Computed tomography; KUB: Kidney ureter bladder.

Plain X-ray of KUB did not show any radio-opaque shadows. Computerized tomography (CT) scan of KUB revealed only bladder calculi (Figures 2, 3).

**Figure 4:**
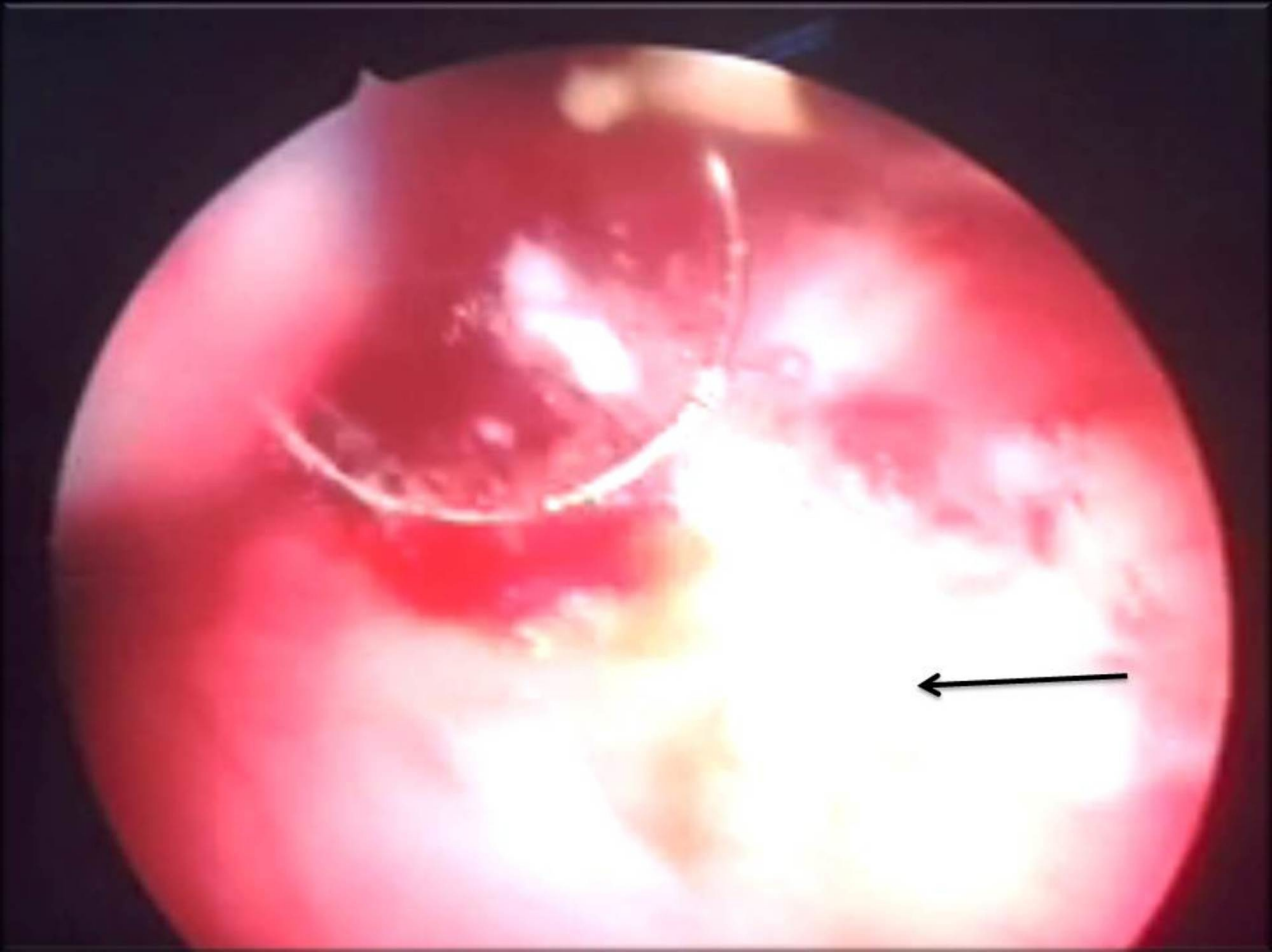
Cystoscopy - Revealed three spiky calculi in the bladder (arrow).

Cystoscopy revealed three spiky calculi in the bladder (Figure 4).

**Figure 5:**
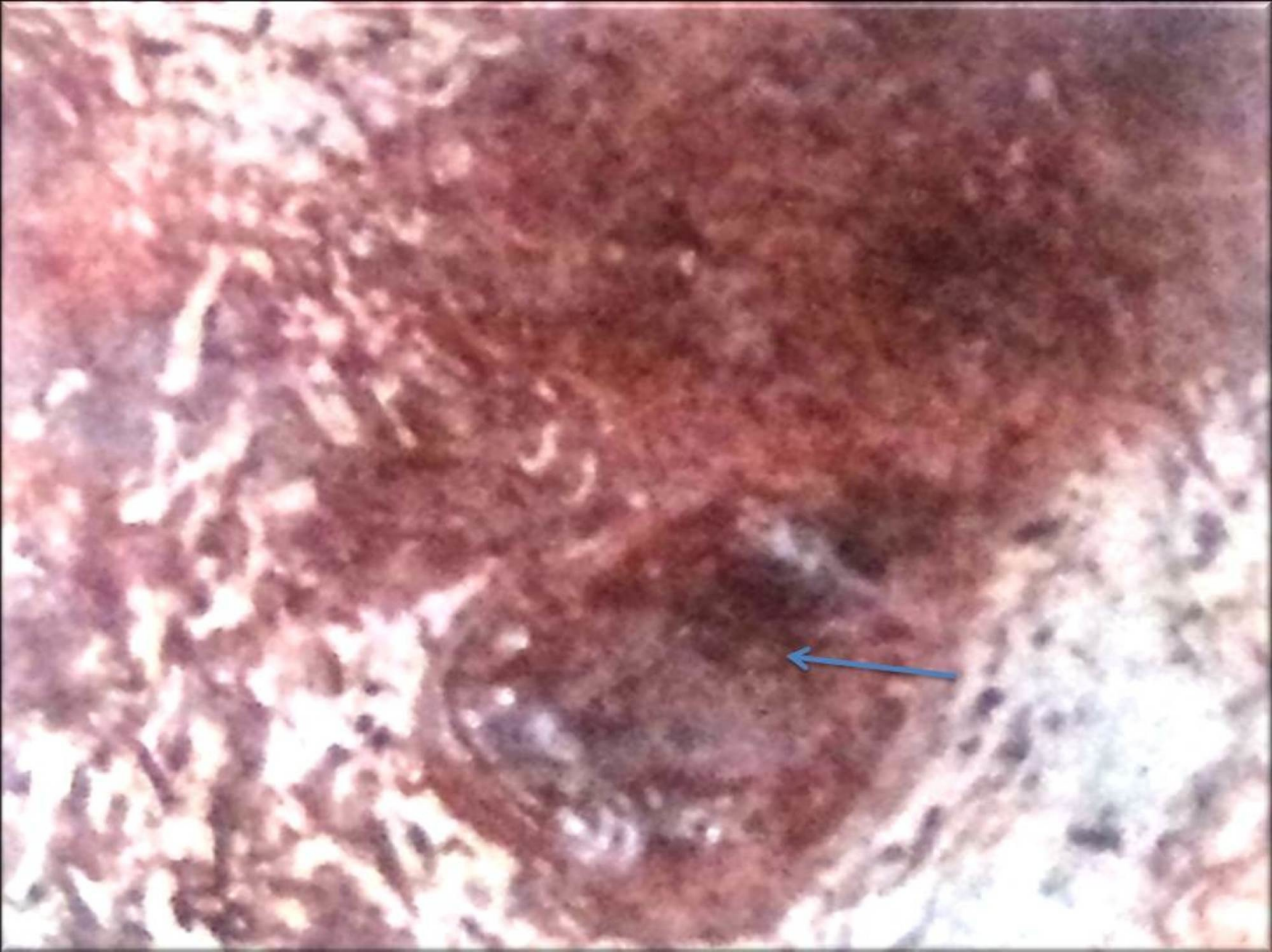
Ureteroscopy (biopsy site) - Fluffy lesion with mucosal edema over the right lateral wall in the region of the right ureteric orifice (arrow).

On ureteroscopy, there was a fluffy lesion with mucosal edema over the right lateral wall in the region of the right ureteric orifice, which was biopsied (Figure 5).

**Figure 6:**
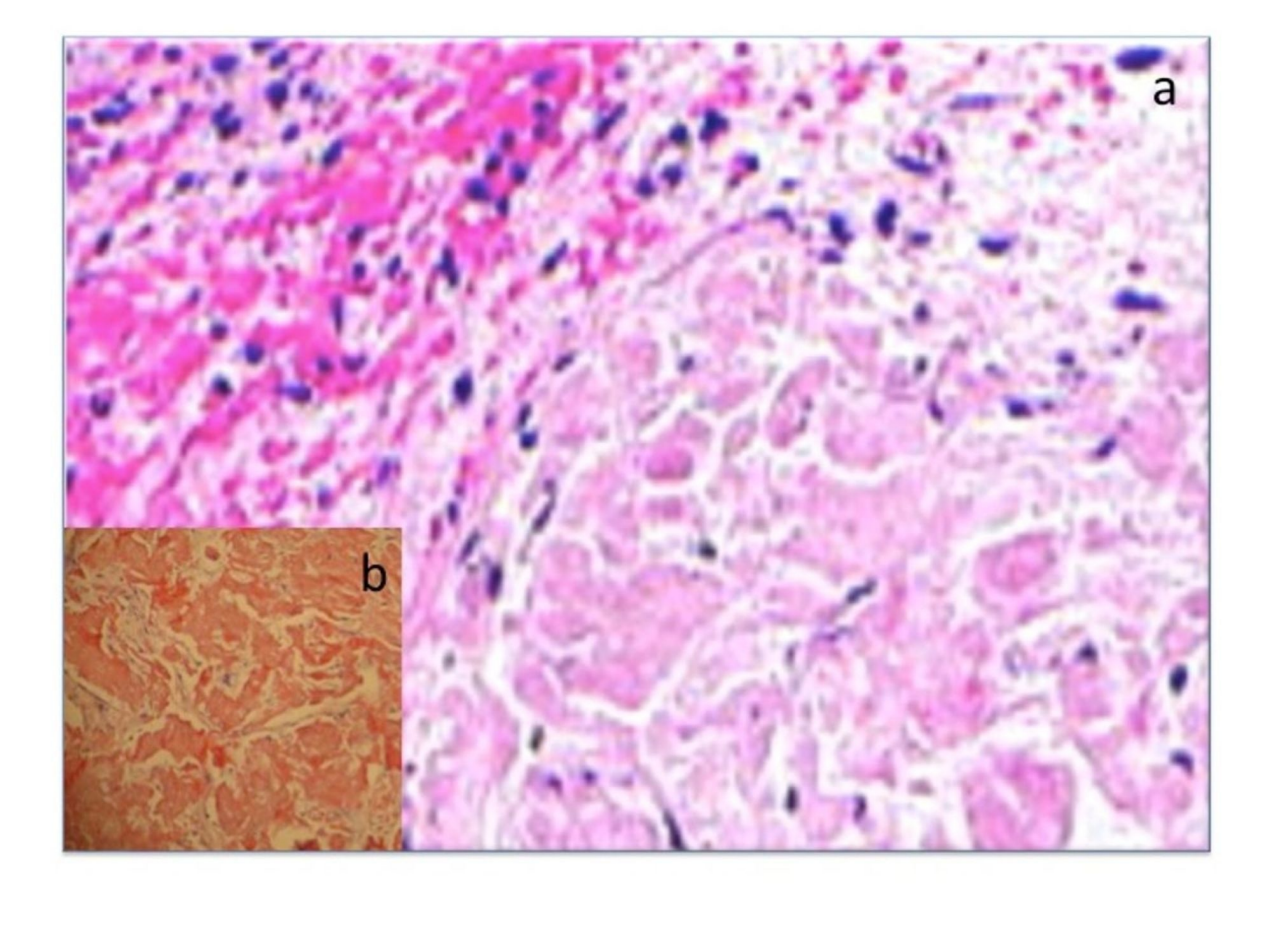
Histopathology slide report confirmed bladder amyloidosis. a) H&E stain. b) Congo red stain. H&E: Hematoxylin and eosin

Biopsy showed fragments of urothelial mucosa with focal areas of ulceration. The underlying stroma was edematous with amorphous pale eosinophilic acellular deposits. Congo red stain showed apple-green birefringence under polarised microscopy suggestive of amyloid. Stroma showed dense infiltrate of plasma cells, lymphocytes and eosinophils. The sections were negative for dysplasia, granulomas or malignancies. Urine routine examination and cytology did not reveal any amyloid crystals. Urine culture was also sterile. Systemic amyloidosis, malignancies and other inflammatory causes also had been ruled out by contrast-enhanced CT abdomen and pelvis. Non-specific stain for amyloidosis like eosin and hematoxylin stain had shown the presence of amyloidosis. Special stain like Congo red stain had confirmed bladder amyloidosis (Figure 6).

After transurethral resection of entire lesion, the patient went home without any major postoperative issues. The patient was called for follow-up imaging and cystoscopy after six months.

## Discussion

Amyloidosis involving in the urinary bladder was first described by Solomin in 1897 [5]. Until 1993, only 93 cases have been reported so far [5]. Primary amyloidosis (PA) of the urinary tract is a rare condition with less than 200 cases reported until the date [6]. The aetiology of this type of PA is unknown. In these cases, urinary bladder wall will be thickened and mucosa will be hyperaemic, rough and nodular with small ulcerations. These pathological changes resemble sessile carcinoma on cystoscopic examination.

The kidney is nearly always involved in secondary amyloidosis and in approximately 50% of the cases of PA [7]. This is in contrast to the urinary bladder, which is usually affected in primary localized amyloidosis [8]. Although rare, it is one of the differential diagnosis in patients with irritative voiding symptoms. In elderly patients, chronic systemic etiology should be checked. Since most cases of VA appear to be of primary type, it is also called as tumor-forming amyloidosis.

Localized amyloidosis poses a diagnostic challenge because of its nonspecific presenting complaints. It may mimic carcinoma or inflammatory pathology, because of its different findings in cystoscopic as well as radiological appearances. Histopathological examination with special staining is necessary to confirm the diagnosis of amyloidosis. It is also required to exclude malignant status [8,9].

Medical treatments like intravesical dimethyl sulfoxide injection and oral colchicines have also been used with limited success rate [10]. Conservative management is transurethral resection of the entire lesion. After the surgical procedure, monthly follow-up is needed for recurrences. The recurrence rates following surgical procedure are 50% [10]. Long-term follow-up with imaging and cystoscopy is required to identify the multiple recurrences.

## Conclusions

Primary VA should also be considered in the evaluation of irritative voiding symptoms. Localized amyloidosis poses a diagnostic challenge because of its nonspecific presenting complaints. It may mimic carcinoma or inflammatory pathology, because of its different findings in cystoscopic as well as radiological appearances.

## References

[1] Singh I, Joshi M, Singh UR, Khan N (2011). Vesical amyloidosis masquerading as bladder cancer with hematuria. Nephro Urol Mon.

[2] Chitale S, Morsey M, Peat D, Webb R (2007). Amyloidosis of lower genitourinary tract: a review. EAU-EBU Update Series.

[3] Altwairgi A (2011). Primary amyloidosis of the urinary bladder presenting as painless haematuria. Int J Health Sci (Qassim).

[4] Raja K, Ahmed E, Mubarak M, Iqbal T, Hassan SM (2013). Primary localized amyloidosis of urinary bladder: a case report and review of literature. Nephro Urol Mon.

[5] Auge BK, Haluszka MM (2000). Primary amyloidosis of the bladder. J Urol.

[6] Merrimen JL, Alkhudair WK, Gupta R (2006). Localized amyloidosis of the urinary tract: case series of nine patients. Urology.

[7] Jain M, Kumari N, Chhabra P, Gupta RK (2008). Localized amyloidosis of urinary bladder: a diagnostic dilemma. Indian J Pathol Microbiol.

[8] Salahia MG, Kebbe Y, Arora A, Pinto T, Hammadeh MY (2014). Primary bladder amyloidosis mimicking bladder malignancy: a case report and literature review. Urol Nephrol.

[9] Paraskevas K, Anagnostou D, Bouris C (2004). Anurea caused by primary amyloidosis of the lower third of the ureters, the ureterovesical junction and the urinary bladder: a case report and review of the literature. Int Urol Nephrol.

[10] Wilkinson M, Fanning DM, Flood H (2011). Primary bladder amyloidosis. BMJ Case Rep.

